# Liver histological study of patients with chronic hepatitis B virus infection in the grey zone

**DOI:** 10.1186/s12879-025-10790-0

**Published:** 2025-03-19

**Authors:** Weijia Lin, Rongrong Ding, Shuangshuang Sun, Wei Lu, Yanbin Wang, Xinlan Zhou, Dan Huang, Xiufen Li, Zhanqing Zhang, Liang Chen

**Affiliations:** 1 Department of Hepatobiliary Medicine, Shanghai Public Health Clinical Center, Fudan University, Shanghai, China; 2 Department of Hepatology Center, Shanghai Public Health Clinical Center, Fudan University, Shanghai, China; 3 Department of Liver Disease, Shanghai Public Health Clinical Center, Fudan University, Shanghai, China

**Keywords:** Chronic HBV infection, Grey zone, Liver biopsy, Significant liver histological changes

## Abstract

**Background and aim:**

The natural history of chronic hepatitis B virus (HBV) infection is usually divided into four phases: immune tolerant (IT), immune active (IA), immune carrier (IC), and immune reactive (IR). Many patients still cannot be classified into the four phases, called “Grey Zone (GZ)”. This study aimed to analyze the liver histological features of the GZ patients to guide antiviral therapy.

**Methods:**

We retrospectively analyzed the 1454 patients with chronic HBV infection who underwent liver biopsy. GZ patients with identical serum hepatitis Be antigen (HBeAg) and alanine aminotransferase (ALT) levels as those in the IT, IA, IC, and IR phases were categorized into the IT-GZ, IA-GZ-1, IA-GZ-2, IC-GZ, and IR-GZ groups, respectively. We analyzed and compared the histological distribution of liver in these patients. We evaluated independent influencing factors for significant liver histological changes (SLHC) in patients in the GZ subgroups.

**Results:**

Among the 1454 patients, 690(47.5%) patients in GZ. Among the 690 patients of the GZ, 322(46.7%) patients for whom histological examinations indicated SLHC. The proportion of SLHC within the GZ subgroups was as follows: IT-GZ (50.5%), IA-GZ-1 (75.0%), IA-GZ-2 (48.4%), IC-GZ (32.1%), and IR-GZ (59.6%). In the IT-GZ group, the proportion of patients aged ≤ 30 years with SLHC was 47.1%, and in the IC-GZ group, this proportion was 42.1%.

**Conclusions:**

46.7% of GZ patients had significant liver histological changes. For HBeAg-negative patients with ALT ≤ 40U/L, HBV DNA ≥ 2000IU/mL, and an age of ≤ 30 years old, antiviral therapy was recommended; if they expressed concern, a liver biopsy was suggested.

**Supplementary Information:**

The online version contains supplementary material available at 10.1186/s12879-025-10790-0.

## Introduction

Hepatitis B virus (HBV) infection is a serious public health issue threatening human health. In 2019, WHO estimated that 296 million people were living with chronic HBV infection, of which only 10% had been diagnosed and 2% had been treated [1]. In regions with high HBV prevalence such as the Asia-Pacific region, more than 50% of hepatocellular carcinomas (HCC) are caused by HBV [2]. Patients receiving antiviral therapy have a 40–60% lower risk of developing HCC compared to untreated patients [3]. Identifying the stages of chronic HBV infection is essential for guiding patient treatment and prognosis.

The natural history of HBsAg-positive chronic HBV infection is typically categorized into four phases: HBeAg-positive chronic HBV infection, previously termed “immune tolerant, (IT)” phase; HBeAg-positive chronic hepatitis B, previously termed “immune active, (IA)” phase; HBeAg-negative chronic HBV infection, previously termed “inactive carrier, (IC)” phase; HBeAg-negative chronic hepatitis B, previously termed “immune reactive, (IR)” phase [4–6]. However, a considerable number of patients with chronic HBV infection still cannot be classified into any of the four phases, called “Grey Zone(GZ)”. Different guidelines have varying serological criteria for natural history phases, resulting in an unclear definition of the GZ [4–6]. However, regardless of the guideline definition of patients with GZ, multiple studies have shown that a higher proportion of patients with GZ have significant liver histological changes [7–10]. The GZ has high heterogeneity, with different combination patterns of HBeAg status, alanine aminotransferase (ALT) and HBV DNA levels, which requires personalized management [11]. Deciding to start antiviral therapy is controversial for GZ patients with different HBeAg statuses. It can be challenging to make an accurate decision without the results of a liver biopsy [12, 13]. Conducting histological studies on patients in the GZ is essential for guiding the treatment of various GZ subgroups.

## Methods

### Patients

A total of 3454 patients with chronic HBV infection who underwent liver biopsy in Shanghai Public Health Clinical Center from January 2010 to July 2022 were selected, and 1454 patients who met the criteria were finally included. Serum HBeAg, HBV DNA, and ALT levels were monitored a minimum of three times within the year preceding the liver biopsy, with follow-up intervals of three months.

A liver biopsy was recommended in patients with chronic hepatitis B to assess the degree of liver inflammation and fibrosis to assist in deciding whether to initiate antiviral therapy. Liver biopsy exclusion criteria: Uncooperative patient; Severe coagulopathy; Infection of the hepatic bed; Extrahepatic biliary obstruction; Ascites; Morbid obesity; Possible vascular lesions; Amyloidosis; Hydatid disease [14]. Informed consent was obtained from all patients before liver biopsy. Laboratory and demographical data were collected within one week prior to liver biopsy.

**Fig. 1:**
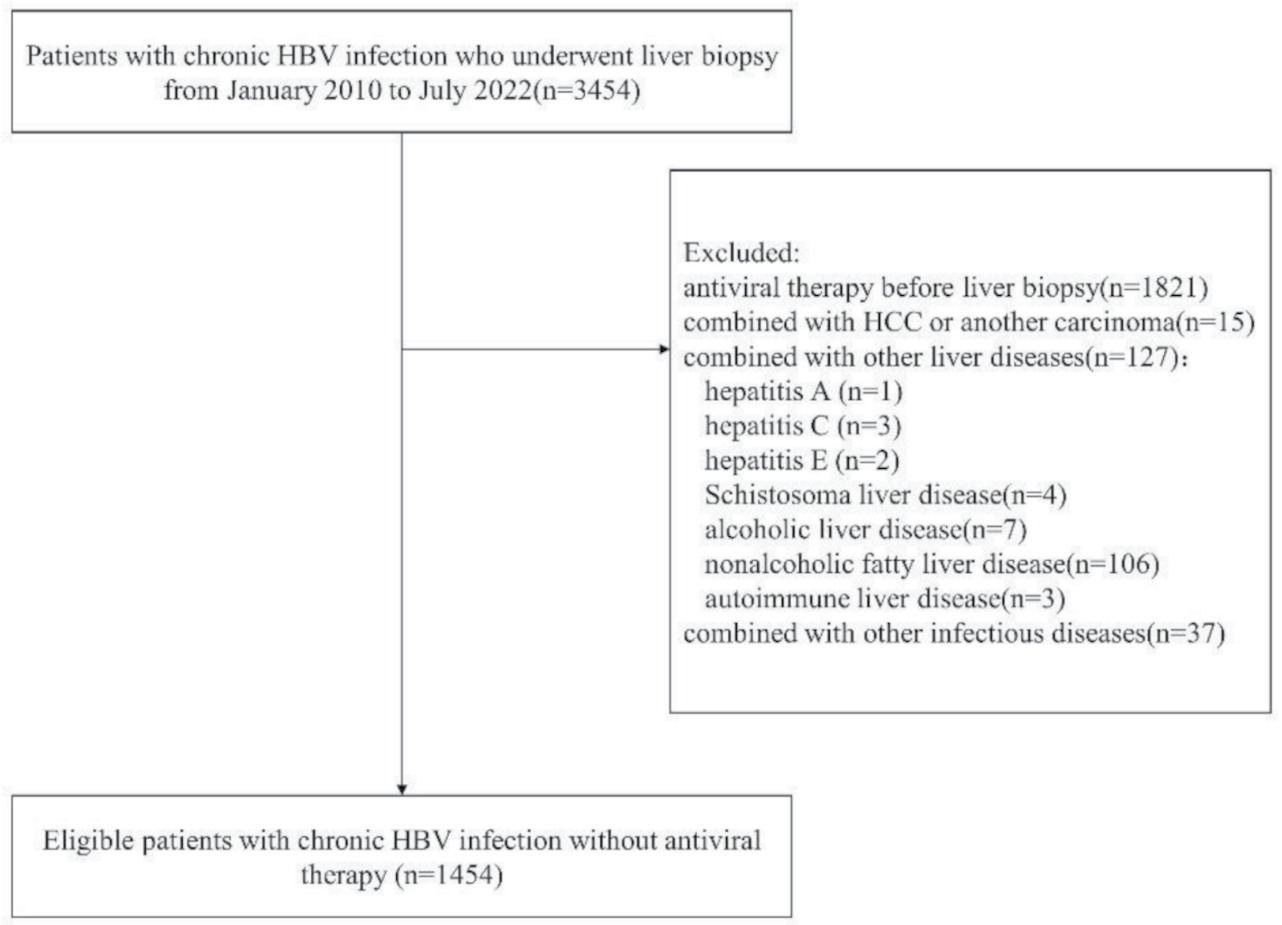
Flow chart of patient selection

The flow chart of patient selection is presented in Fig. 1. Inclusion criteria: (a) HBsAg positive > 6 months. (b) underwent liver biopsy. Exclusion criteria: (a) antiviral therapy before liver biopsy; (b) combined with Hepatocellular carcinoma (HCC) or another carcinoma; (c) combined with other chronic liver diseases such as other hepatitis viruses (hepatitis A, C, D, and E), Schistosoma liver disease, alcoholic liver disease, nonalcoholic fatty liver disease, and autoimmune liver disease; (d) combined with other infectious diseases [tuberculosis, syphilis, and human immunodeficiency virus (HIV)].

**Fig. 2:**
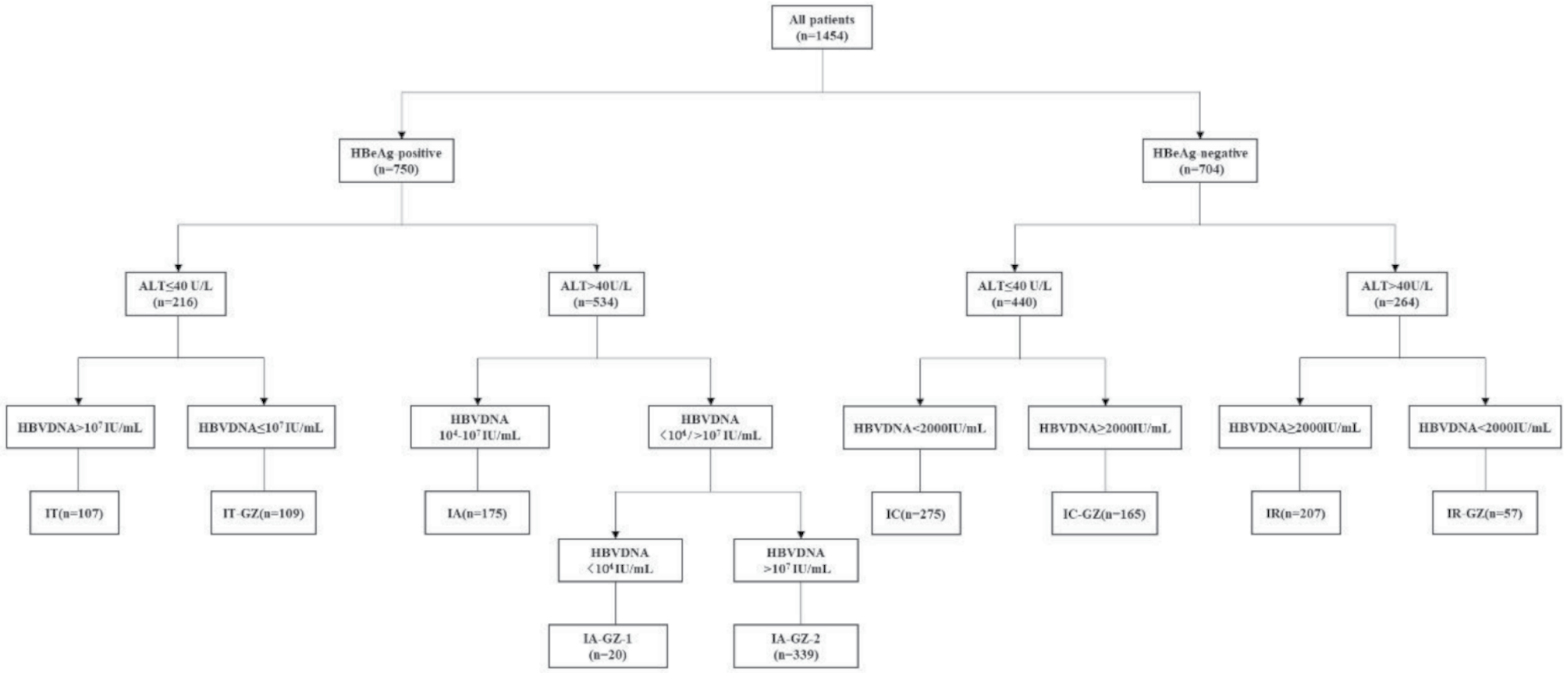
Grouping of patients with chronic HBV infection

According to the European Association for the Study of the Liver (EASL) 2017 Clinical Practice Guidelines, the grouping was based on HBeAg status, ALT levels, and HBV DNA serum levels (Fig. 2) [6]. In patients with HBeAg-positive and ALT ≤ 40U/L, HBV DNA > 10^7^ IU/mL was the IT phase, and HBV DNA ≤ 10^7^ IU/mL was the IT-GZ group. In patients with HBeAg-positive and ALT > 40U/L, HBV DNA between 10^4^ and 10^7^ IU/mL was IA phase, HBV DNA < 10^4^ IU/mL was IA-GZ-1 group, and HBV DNA > 10^7^ IU/mL was IA-GZ-2 group. In patients with HBeAg-negative and ALT ≤ 40 U/L, HBV DNA < 2000 IU/mL was the IC phase, and HBV DNA ≥ 2000 IU/mL was the IC-GZ group. In patients with HBeAg-negative and ALT > 40 U/L, HBV DNA ≥ 2000 IU/mL was the IR phase, and HBV DNA < 2000 IU/mL was the IR-GZ group.

### Histological assessment

All liver biopsy specimens were read by two experienced liver pathologists. The pathologists were blinded to the clinical data of the patients. Liver pathological diagnosis refers to Scheuer scoring system: pathological grade represents the degree of necrotizing inflammation, divided into G0 ∼ G4 five grades; pathological stage represents the degree of fibrosis and structural changes, divided into S0 ∼ S4 five stages. This study defines pathological grade ≥ G2 or/and stage ≥ S2 as significant liver histological changes (SLHC) [15, 16].

### Statistical analysis

Data analysis and graphics were performed using SPSS 26.0 and GraphPad Prism 9 statistical software. For continuous variables, normality testing is performed. If the data follows a normal distribution, it is expressed as mean ± standard deviation, and an independent t-test is used to compare the means. If the data does not follow a normal distribution, it is expressed as median (P25–P75), and the Mann-Whitney test is used. Enumeration data were expressed as the constituent ratio (%); the Pearson χ2 test or Fisher exact test was used to compare groups. Matched data were assessed using paired t-test or Wilcoxon rank sum test. Binary logistic regression analysis was used to identify factors associated with SLHC. *P* < 0.05 was defined as statistically significant.

## Results

### Clinical characteristics of patients with chronic HBV infection among different immune phases and GZ subgroups

Among the 1454 patients, there were 107(7.4%) patients in IT phase, 175(12.0%) patients in IA phase, 275(18.9%) patients in IC phase, 207(14.2%) patients in IR phase, and 690(47.5%) patients in GZ (Table S1). Among patients in the GZ group, the median serum HBV DNA level was 7.0(3.8–7.7) log_1_₀ IU/mL. The relatively broad inter-quartile range implies substantial variability among individuals. We conducted further analysis of the clinical characteristics of the GZ subgroups (Table 1). Among 690 GZ patients, there are 109 (15.8%), 20 (2.9%), 339 (49.1%), 165 (23.9%), and 57 (8.3%) cases in the IT-GZ, IA-GZ-1, IA-GZ-2, IC-GZ, and IR-GZ groups, respectively. Patients in the HBeAg-negative GZ subgroups (IC-GZ and IR-GZ groups) had a higher median age than those in the HBeAg-positive GZ subgroups (IT-GZ, IA-GZ-1, and IA-GZ–2 groups). All subgroups in GZ were predominantly male.

**Table 1:** Characteristics of chronic HBV infection patients in GZ subgroups

Characteristics	GZ (*n* = 690)	IT-GZ (*n* = 109)	IA-GZ-1 (*n* = 20)	IA-GZ-2 (*n* = 339)	IC-GZ (*n* = 165)	IR-GZ (*n* = 57)
Age (years)	34(29–43)	35(29–43)	35(27–46)	30(27–35)	41(35–51)	42(35–49)
Male (%)	430(62.3)	60(55.0)	11(55.0)	224(66.1)	93(56.4)	41(71.9)
ALT(U/L)	52(27–124)	25(19–33)	83(42–136)	98(64–210)	25(18–31)	74(54–139)
AST(U/L)	36(25–70)	24(20–31)	50(33–97)	59(39-113.3)	23(20–27)	48(33–87)
ALP(U/L)	73(60–91)	67(56–87)	90(71–128)	76(62–92)	69(58–84)	834(58–110)
γ-GT (U/L)	27(17–54)	23(14–34)	50(24–130)	33(20–62)	18(14–25)	68(28–114)
CHE(U/L)	7833(6672–9358)	7527(6528–9234)	6310(3119–8016)	7829(6599–9087)	8435(7253–10050)	7756(5881–9667)
TBil(µmol/L)	13.7(10.1–18.6)	12.3(9.5–16.9)	22.5(12.0-61.4)	13.7(10.4–18.4)	13.2(9.6–17.6)	16.5(12.9–22.7)
ALB(g/L)	43.0(40.7-45.43)	42.9(40.6–45.4)	40.3(32.5–43.9)	42.8(40.6–45.0)	44.4(42.0-46.1)	42.9(39.0-45.6)
TBA(µmol/L)	7.6(4.1–14.6)	8.8(4.9–15.5)	27.8(8.1–94.0)	8.8(4.4–15.7)	5.2(3.1–8.8)	8.0(4.3–26.5)
WBC(10^9^/L)	5.2(4.4–6.2)	5.4(4.2–6.4)	5.2(4.0-6.5)	5.2(4.4–6.1)	5.2(4.3–6.3)	5.4(4.5–6.6)
RBC(10^12^/L)	4.66 ± 0.02	4.47(4.23-5.00)	4.29(4.00-4.77)	4.77(4.38–5.11)	4.63(4.23–4.95)	4.62(4.15-5.00)
PLT(10^9^/L)	168(139–205)	156(130–188)	138(104–191)	178(146–208)	168(140–212)	145(113–196)
PT(s)	13.6(13.1–14.2)	13.7(13.2–14.4)	13.8(13.0-14.6)	13.6(13.1–14.3)	13.5(13.0-14.1)	13.7(13.3–14.4)
APTT(s)	38.3(36.60–40.8)	38.7(36.9–41.2)	39.5(37.8–41.7)	38.1(35.6–40.9)	38.1(35.8–40.6)	38.2(35.2–40.5)
HBsAg(log_10_ IU/ml)	3.8(3.1–4.4)	3.4(2.9–3.8)	3.4(2.8-4.0)	4.4(3.9–4.7)	3.3(2.7–3.7)	2.9(1.7–3.2)
HBeAg(positive)[*n* (%)]	468(67.8)	109(100.0)	20(100.0)	339(100.0)	0(0.0)	0(0.0)
HBVDNA (log_10_ IU/mL)	7.0(3.8–7.7)	5.2(3.4–6.3)	3.3(2.9–3.7)	7.7(7.5-8.0)	4.0(3.7–4.8)	2.70(2.69–2.75)
Inflammation [*n* (%)]						
G0-1	486(70.4)	74(67.9)	8(40.0)	224(66.1)	145(87.9)	35(61.4)
G2	161(23.3)	27(24.8)	9(45.0)	90(26.5)	16(9.7)	19(33.3)
G3	43(6.2)	8(7.3)	3(15.0)	25(7.4)	4(2.4)	3(5.3)
G4	0(0.0)	0(0.0)	0(0.0)	0(0.0)	0(0.0)	0(0.0)
Fibrosis [*n* (%)]						
S0-1	399(57.8)	58(53.2)	6(30.0)	194(57.2)	114(69.1)	28(49.1)
S2	187(27.1)	26(23.9)	6(30.0)	102(30.1)	36(21.8)	16(28.1)
S3	52(7.5)	11(10.1)	5(25.0)	23(6.8)	9(5.5)	4(7.0)
S4	52(7.5)	14(12.8)	3(15.0)	20(5.9)	6(3.6)	9(15.8)
SLHC (G ≥ 2 and/or S ≥ 2) [*n* (%)]	322(46.7)	55(50.5)	15(75.0)	164(48.4)	53(32.1)	34(59.6)

Abbreviations: HBV, hepatitis B virus; IT-GZ, immune tolerant-grey zone; IA-GZ, immune active-grey zone; IC-GZ, immune carrier-grey zone; IR-GZ, immune reactive-grey zone; GZ, grey zone; ALT, alanine aminotransferase; AST, aspartate aminotransferase; ALP, alkaline phosphatase; γ-GT,γ-Glutamyl Transferase; CHE: cholinesterase; TBil: total bilirubin; ALB, albumin; TBA, total bile acids; WBC, white blood cell; RBC, red blood cell; PLT, platelet; PT, prothrombin time; APTT, activated partial thromboplastin time; HBsAg, hepatitis B surface antigen; HBeAg, hepatitis B e antigen; G, inflammation grade; S, fibrosis stage; SLHC, significant liver histological changes

### Histological distribution of the liver in patients with different immune phases and GZ subgroups

**Fig. 3:**
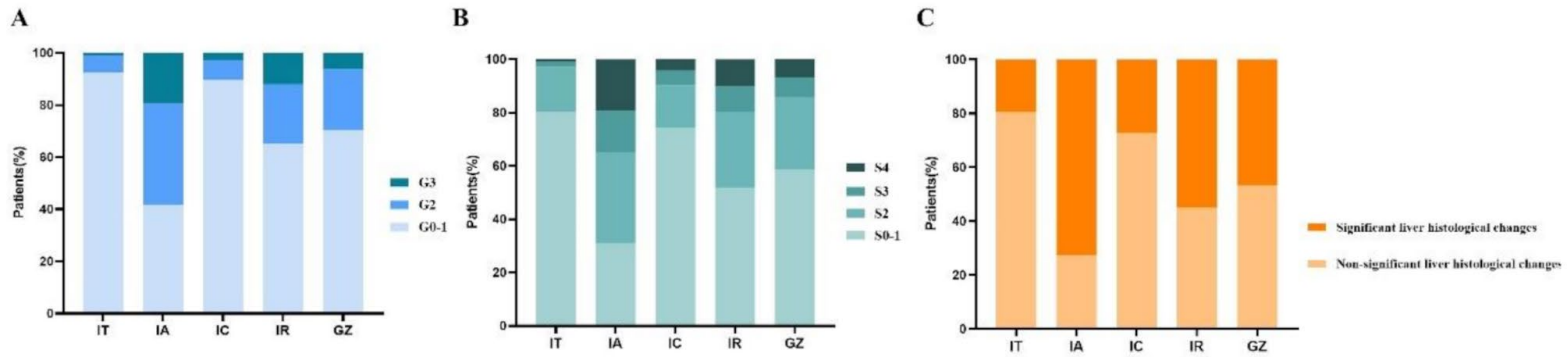
Histological distribution of the liver in patients with different immune phases and GZ. Proportion of liver inflammation grades (**A**), fibrosis stages (**B**), and SLHC (**C**)

The liver histological proportions of the four groups of immune stages and GZ subgroups are shown in Table S1 and Table 1, respectively. The proportion of SLHC was 20/107(18.7%) for IT, 127/175(72.6%) for IA, 75/275 (27.3%) for IC, 114/207 (55.1%) for IR, and 322/690 (46.7%) for GZ. Figure 3 visually shows the liver tissue distribution of patients at different phases. Among patients with inflammation grade ≥ G2, fibrosis stage ≥ S2, and SLHC, the proportion of GZ patients was higher than that of IT and IC phase patients but lower than that of IA and IR phase patients.

**Fig. 4:**
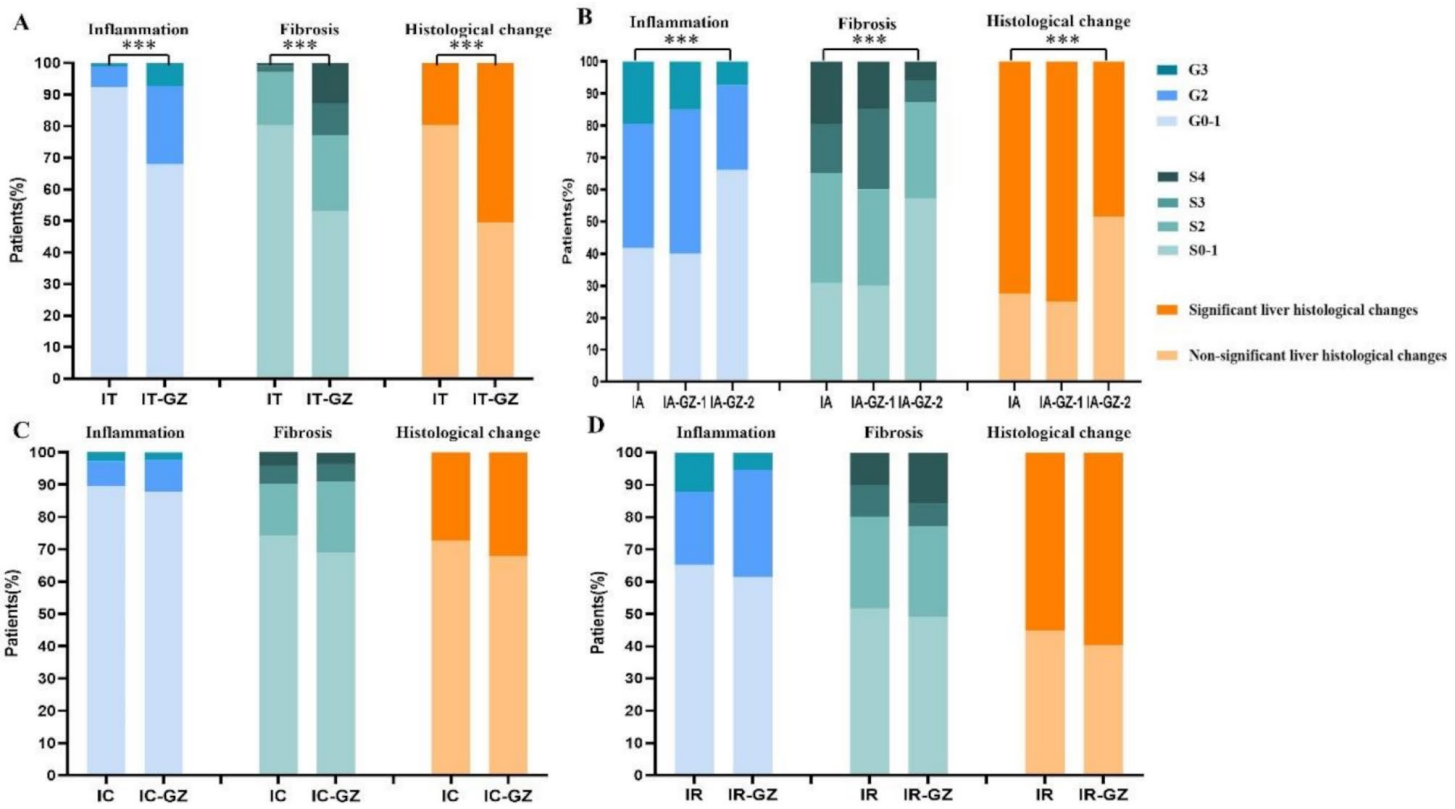
Comparison of liver histological distribution in patients with different immune phases and GZ subgroups. (*A*) Patients with HBeAg-positive and ALT ≤ 40U/L. (*B*) Patients with HBeAg-positive and ALT>40U/L. (*C*) Patients with HBeAg-negative and ALT ≤ 40U/L. (*D*) Patients with HBeAg-negative and ALT>40U/L

The proportion of SLHC in the GZ subgroups was as follows: IT-GZ (50.5%), IA-GZ-1 (75.0%), IA-GZ-2 (48.4%), IC-GZ (32.1%), and IR-GZ (59.6%). The proportion of patients with SLHC in the IT-GZ group was significantly higher than that in the IT phase (50.5% vs. 18.7%, *P* < 0.001) (Fig. 4A). The proportion of patients with SLHC in the IA-GZ-2 group was significantly lower than that in the IA phase (48.4% vs. 72.6%, *P* < 0.001) (Fig. 4B). There was no significant difference in the proportion of SLHC between IR-GZ and IR stage patients (59.6% vs. 55.1%% %, *P* = 0.538) (Fig. 4D).

### The proportion of SLHC in the GZ subgroups was analyzed according to age and sex

**Fig. 5:**
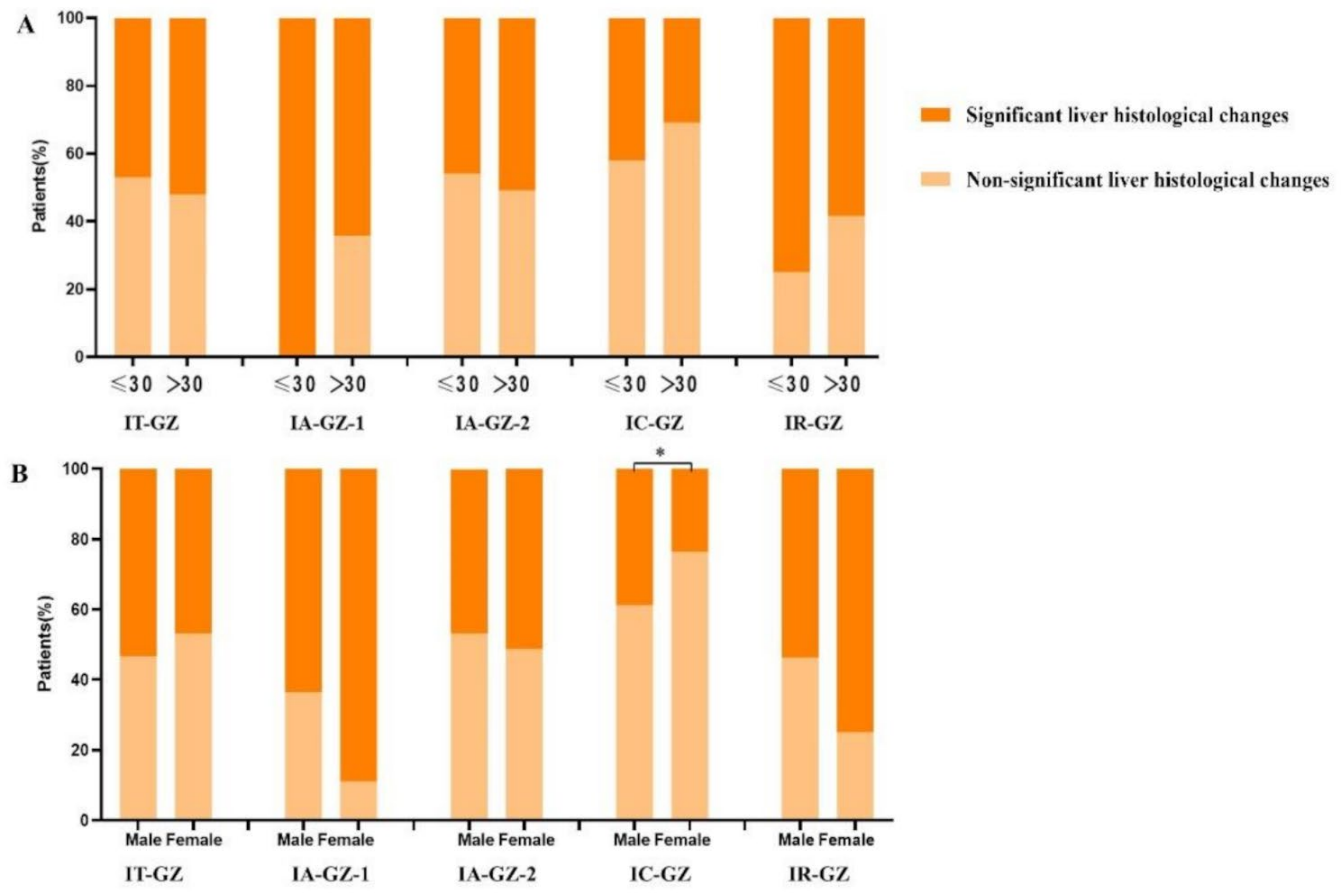
The proportion of SLHC in the GZ subgroups was analysed according to age and sex. (*A*) Subgroup analysis was performed according to age. (*B*)Subgroup analysis was performed according to sex

Subgroup analysis was carried out in five groups of the GZ patients. They were grouped according to whether they were over 30 years old. The proportion of patients over 30 years old with SLHC was higher in the IT-GZ group (≤ 30 years old 47.1% vs. > 30 years old 52.0%, *P* = 0.683) and the IA-GZ-2 group (≤ 30 years old 45.9% vs. > 30 years old 50.9%, *P* = 0.385). In comparison, the proportion of patients under 30 years old with SLHC was higher in the IA-GZ-1 group (≤ 30 years old 100% vs. > 30 years old 64.3%, *P* = 0.260), IC-GZ group (≤ 30 years old 42.1% vs. > 30 years old 30.8%, *P* = 0.433), and IR-GZ group (≤ 30 years old 75.0% vs. > 30 years old 58.5%, *P* = 0.641). None of these groups was statistically significant (Fig. 5A).

These GZ subgroups were also analyzed by sex. The proportion of male patients with SLHC was higher in the IT-GZ group (male 53.3% vs. female 46.9%, *P* = 0.566) and the IC-GZ group (male 38.7% vs. female 23.6%, *P* = 0.045). In comparison, the proportion of female patients with SLHC was higher in the IA-GZ-1 group (male 63.6% vs. female 88.9%, *P* = 0.319), IA-GZ-2 group (male 46.7% vs. female 51.3%, *P* = 0.491), and IR-GZ group (male 53.7% vs. female 75.0%, *P* = 0.229). There was no significant difference in the other four groups except the IC-GZ group (Fig. 5B).

### Independent influencing factors of SLHC in the GZ subgroups

Due to the small number of patients in the IA-GZ-1 group, multivariate analysis was not performed. Multivariate Logistic regression analysis was performed on the remaining four groups of GZ patients (Table 2). In the IT-GZ group, prothrombin time (PT) [odds ratio (OR), 1.909; 95% confidence interval (CI), 1.183–3.078; *P* = 0.008] was an independent predictor of SLHC. The cut-off value of PT was 13.65s, the sensitivity was 67.3%, and the specificity was 60.8%. In the IR-GZ group, albumin (ALB) (OR, 0.847; 95%CI, 0.743–0.964; *P* = 0.012) was an independent predictor of SLHC. The cut-off value of ALB was 40.3 g/L, the sensitivity was 90.9%, and the specificity was 48.5%.

**Table 2:** Multivariate logistic regression analysis of GZ subgroups associated with SLHC

Variables	OR(95%CI)	*P* value
**IT-GZ**		
PT(s)	1.909(1.183–3.078)	0.008
**IA-GZ-2**		
HBVDNA (log_10_ IU/mL)	0.478(0.269–0.848)	0.012
AST(U/L)	1.009(1.005–1.013)	< 0.001
ALB(g/L)	0.927(0.867–0.991)	0.027
**IC-GZ**		
ALP(U/L)	1.030(1.010–1.052)	0.004
ALB(g/L)	0.870(0.777–0.974)	0.016
PLT(10^9^/L)	0.986(0.979–0.994)	< 0.001
**IR-GZ**		
ALB(g/L)	0.847(0.743–0.964)	0.012

NOTE. IT-GZ, grey zone which has the same variable serum levels of HBeAg and alanine aminotransferase as immune tolerant phase (HBeAg-positive, ALT ≤ 40U/L, and HBVDNA ≤ 10^7^ IU/mL); IA-GZ-2: grey zone which has the same variable serum levels of HBeAg and alanine aminotransferase as immune active phase (HBeAg-positive, ALT > 40U/L, and HBVDNA > 10^7^ IU/mL ); IC-GZ, grey zone which has the same variable serum levels of HBeAg and alanine aminotransferase as immune carrier phase (HBeAg-negative, ALT ≤ 40U/L, and HBVDNA ≥ 2000 IU/mL); IR-GZ, grey zone which has the same variable serum levels of HBeAg and alanine aminotransferase as immune reactive phase (HBeAg-negative, ALT > 40U/L, and HBVDNA < 2000IU/mL)

Abbreviations: SLHC, significant liver histological changes; OR: odds ratio; CI, confidence interval; PT, prothrombin time; AST, aspartate aminotransferase; ALB, albumin; ALP, alkaline phosphatase; PLT, platelet.

## Discussion

Chronic HBV infection is a dynamic process that reflects the interaction between HBV replication and host immune response [6]. Identifying the different stages of the natural history of chronic HBV infection is crucial for determining prognosis and guiding treatment [5, 17]. However, recent studies have shown that a large proportion of individuals with chronic HBV infection cannot be classified into any specific phase based on their immune status and are considered to be in the “Grey Zone” of chronic HBV infection [10, 18]. Different guidelines have varying HBV DNA and ALT thresholds, leading to inconsistent criteria for GZ. The prognosis for GZ patients is also inconsistent, and there is still no consensus on their treatment [19]. Antiviral therapy is primarily guided by serum HBV DNA levels, serum ALT levels, and the severity of liver disease [4, 6]. Liver biopsy can assess the severity of liver inflammation and fibrosis; however, its invasive nature limits its widespread use [14]. Therefore, our study analyzed the liver pathology of GZ patients, investigated the risk factors for SLHC, and aimed to guide antiviral treatment in this population.

In our study, 1454 patients with chronic HBV infection were immunologically staged according to EASL natural history serological staging criteria, among which GZ patients accounted for 47.5%. Previous studies have shown that the GZ accounts for about 20–50% [8–11, 18]. This is similar to our research findings. Out of 690 patients with GZ, 322 (46.7%) exhibited SLHC. Some studies have reported a higher proportion of SLHC compared to our findings [8, 9]. Wang et al. conducted a study based on the American Association for the Study of Liver Diseases (AASLD) 2018 hepatitis B guidance, which showed that among 242 GZ patients, approximately 72.7% had SLHC [8]. Gan et al. reported that 75.72% of 696 patients in the GZ, classified according to EASL criteria, exhibited SLHC [9]. Although the GZ criteria in the two studies mentioned above were inconsistent, more than 70% of patients in both studies showed SLHC. Therefore, patients with chronic HBV infection in the GZ require our attention to enhance the clinical understanding of their condition and assess the necessity for antiviral therapy.

Given the heterogeneity among GZ patients with chronic HBV infection, different combinations of HBeAg status, ALT, and HBV DNA levels may have different characteristics. Our study divided GZ patients into five subgroups: IT-GZ, IA-GZ-1, IA-GZ-2, IC-GZ, and IR-GZ. According to the AASLD 2018 Hepatitis B Guidance, Wang et al. categorized GZ patients into four distinct groups: GZ-A, GZ-B, GZ-C, and GZ-D [8]. Because the ALT ULN and HBV DNA level standards of the 2018 guideline and the 2017 guideline of EASL are inconsistent, there are specific differences in the grouping criteria of the GZ. However, the grouping criteria for GZ-A were similar to those for our IT-GZ patients, GZ-B was similar to IA-GZ-1, GZ-C was similar to IC-GZ, and GZ-D was similar to IR-GZ. Wang et al. reported the following proportions of SLHC: GZ-A (84.0%), GZ-B (100.0%), GZ-D (69.9%), and GZ-C (67.0%). In our study, the corresponding values were IT-GZ (50.5%), IA-GZ-1 (75%), IA-GZ-2 (48.4%), IC-GZ (32.1%), and IR-GZ (59.6%). Similar to their study, our IA-GZ-1 patients exhibited the highest proportion of significant histological changes within the group.

In this study, we analyzed HBeAg-positive patients with ALT levels of 40 U/L or lower. The IT-GZ group exhibited a relatively high proportion of SLHC, exceeding 50%. Zhang et al. assessed untreated patients with HBeAg-positive chronic HBV infection and normal ALT levels, discovering significant liver inflammation in those with low mean HBV DNA levels [20]. Duan et al. evaluated 179 HBeAg-positive chronic hepatitis B patients with normal ALT and found that the proportion of SLHC in the HBV DNA < 10^7^ IU/mL group was higher than that in the HBV DNA ≥ 10^7^ IU/mL group (81.8% vs. 54.1%, *P* < 0.05) [7]. Xu et al. analyzed HBeAg-positive patients with normal ALT levels and found that HBV DNA levels were negatively associated with severe fibrosis (*P* < 0.001) [21]. Similarly, our study found that compared to patients in the IT phase with HBV DNA >10^7^ IU/mL, those in the IT-GZ group with lower HBV DNA levels were more likely to exhibit SLHC. According to Chinese guidelines, if histological examinations indicate G < 2 and S < 2, for patients with normal ALT levels and who are over 30 years old, antiviral treatment is recommended [5]. Therefore, we grouped the patients according to whether they were over 30 years old. In the IT-GZ group, the proportion of SLHC patients aged ≤ 30 years was also as high as 47.1%. Therefore, we recommend antiviral treatment for this subset of patients.

We analyzed patients with HBeAg-positive and ALT>40U/L. The proportion of SLHC in the IA-GZ-1 and IA-GZ-2 groups was relatively high, reaching 75% and 48.4% respectively. Thus, we recommend antiviral therapy in these two groups. This is consistent with the latest recommendations from the Chinese chronic hepatitis B guideline (2022 version), which state that antiviral therapy is recommended for patients with detectable HBV DNA and persistently elevated ALT levels (> ULN) after excluding other causes [5]. We found that patients in the IA phase and IA-GZ-1 group with lower HBV DNA levels were more likely to have SLHC than patients in the IA-GZ-2 group with HBV DNA levels greater than 10^7^ IU/mL. In the multivariate analysis of patients in the IA-GZ-2 group, HBV DNA level (OR 0.478, 95%CI 0.269–0.848; *P* = 0.012) was significantly negatively correlated with SLHC. Xie et al. performed liver biopsies on 234 untreated HBeAg-positive chronic hepatitis B patients and found that HBV DNA levels were negatively correlated with significant fibrosis in the mildly abnormal ALT subgroup (*P* = 0.021) [22]. Liao et al. found that HBV DNA negatively correlates with severe inflammatory necrosis and fibrosis in HBeAg-positive patients with elevated ALT [23]. In patients with HBeAg-positive, HBV DNA levels appear to be inversely associated with SLHC.

HBeAg-negative patients with normal ALT were analyzed. Liao et al. found that the incidence of significant fibrosis in such patients was 30.9% [23]. Gao et al. found that more than 40% of such patients had noticeable liver histopathological changes [24]. Gui et al. also found that 25.4% of patients had significant inflammation and/or fibrosis in a retrospective cohort [25]. In our study, the SLHC in our HBeAg-negative patients with normal ALT were about 1/3. The proportion of SLHC in the IC-GZ group with HBV DNA ≥ 2000 IU/mL was 32.1%. Ormeci et al. performed liver biopsies in chronic hepatitis B patients with PNALT and high HBV DNA load (≥ 2000 IU/mL) that were similar to those in our IC-GZ group, with a significant liver injury rate of up to 40%, slightly higher than in our study population [26]. Notably, the proportion of patients with SLHC in the IC-GZ group aged ≤ 30 years reached 42.1%. We advocate for antiviral treatment for this subgroup of patients. If these patients express concerns regarding antiviral therapy, we propose conducting a liver biopsy.

EASL recommends that HBeAg-negative, HBV DNA > 2000 IU/mL, and ALT > upper limit of normal (ULN) (IR phase) should receive treatment [6]. Among our HBeAg-negative patients with abnormal ALT, given that the proportion of SLHC in the IR and IR-GZ groups attained 55.1% and 59.6% respectively, we recommend antiviral treatment. This is also in line with the Chinese chronic hepatitis B guideline (2022 version) [5]. From the perspective of antiviral treatment, the GZ in distinguishing HBeAg-negative patients with abnormal ALT may not be of much significance.

Our study has several limitations. First, as a retrospective study, selection bias may have existed. Second, our study lacks follow-up data, and we will continue to follow up with patients in the GZ in our subsequent studies. Third, as a single-center study, our findings might still require external validation.

In conclusion, 46.7% of the 690 patients in the Grey Zone had histology examinations that indicated SLHC. In particular, 42.1% of patients under 30 years old in the IC-GZ group had SLHC. For patients with chronic hepatitis B in the IT-GZ, IA-GZ-1, IA-GZ-2, and IR-GZ groups, as well as those in the IC-GZ group who were over 30 years of age, we suggested antiviral treatment. In the case of patients under 30 years old in the IC-GZ group, antiviral therapy was also recommended. However, if they expressed concerns about the antiviral treatment, performing a liver biopsy was proposed as an alternative approach for more accurate assessment.

## Electronic supplementary material

Below is the link to the electronic supplementary material.

Supplementary Material 1

## Data Availability

The datasets analysed during the current study are not publicly available due to the privacy of the individuals involved but are available from the corresponding author on reasonable request.

## Abbreviations

AASLD: American Association for the Study of Liver Diseases;
ALB: Albumin
ALP: Alkaline phosphatase
ALT: Alanine aminotransferase
APTT: Activated partial thromboplastin time
AST: Aspartate aminotransferase
CHE: Cholinesterase
CI: Confidence interval
EASL: European Association for the Study of the Liver
G: Inflammation grade
γ-GT: γ-Glutamyl Transferase
GZ: Grey zone
HBeAg: Hepatitis B e antigen
HBsAg: Hepatitis B surface antigen
HBV: Hepatitis B virus
HCC: Hepatocellular carcinomas
HIV: Human immunodeficiency virus
IA: Immune active
IC: Immune carrier
IR: Immune reactive
IT: Immune tolerant
OR: Odds ratio
PLT: Platelet
PT: Prothrombin time
RBC: Red blood cell
S: Fibrosis stage
SLHC: Significant liver histological changes
TBA: Total bile acids
TBil: Total bilirubin
WBC: White blood cell
