# Supplementary material for: Liver histological study of patients with chronic hepatitis B virus infection in the grey zone
Source: BMC Infect Dis. 2025 Mar 19;25:385. doi: 10.1186/s12879-025-10790-0 (PMC11924787; doi:10.1186/s12879-025-10790-0)
Supplement: Supplementary file 1 — Supplementary Material 1 [file 12879_2025_10790_MOESM1_ESM.docx]

**Table S1** Characteristics of chronic HBV infection patients among different immune phases.

| **Characteristics** | **IT**  **(n=107)** | **IA**  **(n=175)** | **IC**  **(n=275)** | **IR**  **(n=207)** | **GZ**  **(n=690)** |
| --- | --- | --- | --- | --- | --- |
| Age (years) | 33(28-39) | 34(27-42) | 42(34-50) | 43(36-50) | 34(29-43) |
| Male (%) | 45(42.1) | 114(65.1) | 160(58.2) | 123(59.4) | 430(62.3) |
| ALT(U/L) | 24(19-31) | 98(57-251) | 20(14-26) | 81(57-162) | 52(27-124) |
| AST(U/L) | 22(20-27) | 63(41-119) | 21(18-24) | 56(38-102) | 36(25-70) |
| ALP(U/L) | 63(54-75) | 81(68-101) | 66(55-80) | 78(65-94) | 73(60-91) |
| γ-GT (U/L) | 15(11-26) | 49(30-92) | 17(12-25) | 40(24-73) | 27(17-54) |
| CHE(U/L) | 7761(6487-9088) | 7520(6026-8564) | 8300(7162-9958) | 7858(6284-10078) | 7833(6672-9358) |
| TBil(μmol/L) | 10.8(8.3-14.5) | 14.5(10.0-20.1) | 13.4(9.2-18.0) | 15.1(11.0-20.6) | 13.7(10.1-18.6) |
| ALB(g/L) | 42.9(40.5-44.9) | 42(39.3-44.1) | 44.1(41.4-46.3) | 43.0(40.0-45.1) | 43.0(40.7-45.43) |
| TBA(μmol/L) | 5.7(2.9-9.2) | 11.0(6.1-23.4) | 4.7(2.7-9.1) | 7.3(3.7-13.4) | 7.6(4.1-14.6) |
| WBC(10^9^/L) | 5.3(4.3-6.1) | 5.5(4.4-6.2) | 5.1(4.2-6.2) | 5.2(4.1-6.2) | 5.2(4.4-6.2) |
| RBC(10^12^/L) | 4.53±0.05 | 4.63±0.04 | 4.57±0.03 | 4.56±0.04 | 4.66±0.02 |
| PLT(10^9^/L) | 175(154-209) | 158(127-192) | 167(131-209) | 157(117-185) | 168(139-205) |
| PT(s) | 13.3(12.9-13.8) | 13.9(13.2-14.4) | 13.5(13.0-14.1) | 13.8(13.2-14.4) | 13.6(13.1-14.2) |
| APTT(s) | 37.1(34.2-30.0) | 38.4(36.5-41.2) | 37.9(35.5-40.1) | 38.1(36.2-40.7) | 38.3(36.60-40.8) |
| HBsAg（log_10_ IU/ml） | 4.7(4.5-4.9) | 3.5(3.1-3.8) | 3.0(2.2-3.6) | 3.3(2.9-3.7) | 3.8(3.1-4.4) |
| HBeAg(positive)[n (%)] | 107(100.0) | 175(100.0) | 0(0.0) | 0(0.0) | 468(67.8) |
| HBVDNA (log_10_ IU/mL) | 7.7(7.7-8.0) | 6.3(5.6-6.6) | 2.7(2.7-2.8) | 5.5(4.5-6.3) | 7.0(3.8-7.7) |
| Inflammation [n (%)] |  |  |  |  |  |
| G0-1 | 99(92.5) | 73(41.7) | 246(89.5) | 135(65.2) | 486(70.4) |
| G2 | 7(6.5) | 68(38.9) | 21(7.6) | 47(22.7) | 161(23.3) |
| G3 | 1(0.9) | 34(19.4) | 8(2.9) | 25(12.1) | 43(6.2) |
| G4 | 0(0.0) | 0(0.0) | 0(0.0) | 0(0.0) | 0(0.0) |
| Fibrosis [n (%)] |  |  |  |  |  |
| S0-1 | 87(81.3) | 54(30.9) | 204(74.2) | 107(51.7) | 399(57.8) |
| S2 | 17(15.9) | 60(34.3) | 44(16.0) | 59(28.5) | 187(27.1) |
| S3 | 2(1.9) | 27(15.4) | 15(5.5) | 20(9.7) | 52(7.5) |
| S4 | 1(0.9) | 34(19.4) | 12(4.4) | 21(10.1) | 52(7.5) |
| SLHC (G≥2 and/or S ≥2)[n (%)] | 20(18.7) | 127(72.6) | 75(27.3) | 114(55.1) | 322(46.7) |

Abbreviations: HBV, hepatitis B virus; IT, immune tolerant; IA, immune active; IC, immune carrier; IR, immune reactive; GZ, grey zone; ALT, alanine aminotransferase; AST, aspartate aminotransferase; ALP, alkaline phosphatase; γ-GT,γ-Glutamyl Transferase; CHE: cholinesterase; TBil: total bilirubin; ALB, albumin; TBA, total bile acids; WBC, white blood cell; RBC, red blood cell; PLT, platelet; PT, prothrombin time; APTT, activated partial thromboplastin time; HBsAg, hepatitis B surface antigen; HBeAg, hepatitis B e antigen; G, inflammation grade; S, fibrosis stage; SLHC, significant liver histological changes.
